# Molecular Epidemiological, Serological, and Pathogenic Analysis of EV-B75 Associated With Acute Flaccid Paralysis Cases in Tibet, China

**DOI:** 10.3389/fmicb.2020.632552

**Published:** 2021-01-13

**Authors:** Keyi Zhang, Mei Hong, Yong Zhang, Zhenzhi Han, Jinbo Xiao, Huanhuan Lu, Yang Song, Dongmei Yan, Dongyan Wang, Shuangli Zhu, Wenbo Xu, Guizhen Wu

**Affiliations:** ^1^WHO WPRO Regional Polio Reference Laboratory, NHC Key Laboratory for Biosafety, NHC Key Laboratory for Medical Virology, National Institute for Viral Disease Control and Prevention, Chinese Center for Disease Control and Prevention, Beijing, China; ^2^Tibet Center for Disease Control and Prevention, Lhasa, China; ^3^Center for Biosafety Mega-Science, Chinese Academy of Sciences, Wuhan, China

**Keywords:** enterovirus B75, seroprevalence, temperature sensitivity, Tibet, phylogenetic analyses

## Abstract

Enterovirus B75 (EV-B75) is a newly identified serotype of the enterovirus B species. To date, only 112 cases related to EV-B75 have been reported worldwide, and research on EV-B75 is still limited with only two full-length genome sequences available in GenBank. The present study reported seven EV-B75 sequences from a child with acute flaccid paralysis and six asymptomatic close contacts in Shigatse, Tibet. Phylogenetic analysis revealed that the Tibetan strain was possibly imported from neighboring India. Seroepidemiological analyses indicated that EV-B75 has not yet caused a large-scale epidemic in Tibet. Similarity plots and boot scanning analyses revealed frequent intertypic recombination in the non-structural region of all seven Tibet EV-B75 strains. All seven Tibetan strains were temperature-sensitive, suggesting their poor transmissibility in the environment. Overall, though the seven Tibetan strains did not cause large-scale infection, prevention and control of the novel enterovirus cannot be underestimated.

## Introduction

Enteroviruses (genus Enterovirus, family Picornaviridae) are divided into 15 species assigned to enteric enterovirus (EV) A–L and respiratory rhinovirus A–C (Walker et al., 2019). Human pathogenic enteroviruses are classified under EV-A–D, including poliovirus (PV) (I–III), coxsackievirus (CV), echovirus, and novel enteroviruses. These diverse serotypes cause a diverse array of clinical features such as aseptic meningitis, hand foot and mouth disease (HFMD), neonatal sepsis-like disease, pancreatitis, encephalitis, myocarditis, pericarditis, acute flaccid paralysis (AFP), and severe respiratory disease. As per the International Committee on Taxonomy of Viruses (ICTV) classification, enteroviruses newly discovered after 1976 are named as novel enteroviruses, namely EV-D68 to the current EV-A121, with a total of 53 genotypes.

Most novel enteroviruses are not the main pathogens of intestinal-transmitted diseases and rarely cause concentrated outbreaks worldwide. However, increasing novel enteroviruses have been found and defined in recent years using innovations in molecular typing methods. Occasionally, a previously rare enterovirus genotype may cause large-scale outbreaks or continuous epidemics. EV-A71, a member of the novel enterovirus family, was defined in 1974. Following the outbreak of HFMD caused by EV-A71 infection in Linyi, Shandong Province, in 2007 (Zhang et al., 2009) and in Fuyang, Anhui Province, in 2008 (Zhang et al., 2010), laboratory surveillance of HFMD was conducted nationwide in China. Since then, the reported cases of EV-A71 have increased annually, resulting in EV-A71 being the predominant HFMD pathogen and one of the leading pathogens for severe and fatal cases of HFMD. EV-D68, another rarely reported enterovirus, caused an endemic outbreak of severe respiratory illness in the United States, with more than 1,150 laboratory-confirmed cases (Greninger et al., 2015). Thus, comprehensive surveillance and precise investigation of viral prevalence and transmission is critical for the control and intervention of enterovirus diseases.

Currently, based on the novel enterovirus isolates available in GenBank database, most novel enterovirus genotypes except for EV-D68 and EV-A71 have been isolated in the mainland of China, and the clinical manifestation of the vast majority of cases related to novel enterovirus is AFP (Oberste et al., 2004; Bingjun et al., 2008; Tao et al., 2010, 2013, 2014a; Xu et al., 2011a,b; Sun et al., 2013; Hu et al., 2014; Liu et al., 2014; Lu et al., 2014; Tang et al., 2014; Zhang et al., 2014, 2016; Fan et al., 2015; Song et al., 2017; Han et al., 2018; Huang et al., 2018; Wang et al., 2019). A considerable number of strains have been isolated from healthy people (Wu et al., 2013; Hu et al., 2014; Tao et al., 2014b; Fan et al., 2015; Tang et al., 2016; Zhang et al., 2016; Song et al., 2017; Han et al., 2018; Zhang et al., 2019), directly indicating that the population carries the novel enteroviruses and is at risk of developing the related diseases. Of these, a large number of cases arising from some novel enterovirus serotypes have already been reported in China, such as EV-B85 in Xinjiang (Sun et al., 2013) (33 cases, 2011, AFP and his asymptomatic contacts), EV-A90 in Xinjiang (Huang et al., 2018) (5 cases, 2011, AFP) and Shandong (Tao et al., 2013) (3 cases, 2001, AFP), EV-C96 in Yunnan (Bingjun et al., 2008) (18 cases, 1999-2010, AFP), and Jiangxi Province (13 cases, 2016, unknown), and EV-C104 in Shandong (Xiang et al., 2013) (five cases, 2011–2012, lower respiratory tract infection). However, due to the lack of full-length genome sequences and detailed epidemiological information, the study of novel enteroviruses has not attracted sufficient attention.

Enterovirus B75 (EV-B75) is a novel enterovirus serotype within EV-B, identified in 2004 (Oberste et al., 2004). In this study, we report the isolation of EV-B75 from a case of AFP and six asymptomatic close contacts from Shigatse, Tibet, China, in 2007. We determined the full-length genomic sequence of these seven Tibetan isolates, and used phylogenetic analysis to track the infection source and to determine recombination events incorporating sequences from the current outbreaks, from public databases. Serum neutralization tests were used to evaluate the population prevalence of EV-B75 in Tibet, and temperature sensitivity tests were used to determine the environmental transmission capacity of the seven strains.

## Materials and Methods

### Sample Collection

The collection, use, and analysis of all samples involved in this study were approved by the Ethics Review Committee of the National Institute for Viral Disease Control and Prevention, Chinese Center for Disease Control and Prevention. Written informed consent for the use of their clinical samples was acquired from the parents of all the children included in this study. All experimental protocols were approved by the National Institute for Viral Disease Control and Prevention, and the methods were carried out in accordance with the approved guidelines.

Seven strains of EV-B75 were isolated from the feces of a patient with AFP and his close contacts during an epidemiological investigation in Shigatse, Tibet, by the Center for Disease Control and Prevention of Tibet Autonomous Region in 2007. All children from which the EV-B75 virus had been isolated, were under 5 years of age. We named the seven EV-B75 Tibetan strains as Y16/XZ/CHN/2007, Y17/XZ/CHN/2007, Y18/XZ/CHN/2007, Y20/XZ/CHN/2007, Y24/XZ/CHN/2007, Y25/XZ/CHN/2007, and Y26/XZ/CHN/2007, hereafter abbreviated as Y16, Y17, Y18, Y20, Y24, Y25, and Y26. In addition, 112 serum samples from Tibetan children used in this study were also collected by the Tibet Autonomous Region Center for Disease Control and Prevention from December 2010 to January 2011, and sent to the National Polio Reference Laboratory for monitoring antibody levels after polio vaccination. All samples were collected from healthy children (aged 1–14 years) in Tibet, including 36 from Shigatse and 76 from Lhasa.

### Virus Isolation and Plaque Purification

All collected fecal specimens were pre-treated according to the standard operating method of the National Polio Reference Laboratory to obtain fecal suspensions, which were then, respectively, inoculated onto human rhabdomyosarcoma (RD) cells for continuous culture for three generations to isolate the virus. The cell line was provided by the WHO Global Poliovirus Specialized Laboratory in the United States and was originally purchased from the American Type Culture Collection (Manassas, VA, United States). When a complete EV-like cytopathic effect (CPE) was observed, the cell cultures were harvested.

All seven viral strains (Y16, Y17, Y18, Y20, Y24, Y25, and Y26) were further purified using plaque tests and then amplified on RD cells. Briefly, RD cells were cultured in a 6-well plate. After the cells were confluent, 1 ml of virus solution diluted 10^5^ times was added and the cells were incubated for 1.5 h (36°C, 5% CO_2_). The 6-well plate was slightly shaken every 30 min to avoid accumulation of the virus solution. Next, a 2.8% low melting point agarose (melting point 65°C) solution, sterilized at high temperature and mixed with the same amount of 2x MEM to form a maintenance medium, was slowly poured into the culture to fix the virus, and the plate was then cultured in an inverted position. On the next day, neutral red dye solution was added, followed by culturing for 2–8 days. When spots were observed, they were picked and further amplified on RD cells.

### Molecular Typing and Full-Length Genome Sequencing

We used Primscript One-Step RT-PCR Kit Ver.2 (Takara, Dali, China) for RNA fragment amplification. The VP1 region sequence was amplified by using primers E490/E492 (E490: TGIGTIYTITGYRTICATTGGAT, E492: GGRTTIGTIGWYTGCCA) (Oberste et al., 2006), and the full-length genomic sequence was amplified by the primers designed by the only two full-length genome sequences in GenBank.

The PCR reaction process was divided into three stages: the first stage was a reverse transcription stage, which was maintained at 50°C for 30 min to reverse transcribe RNA into cDNA, and at 94°C for 2 min to inactivate the reverse transcriptase; the second stage was “denaturation-annealing-extension” amplification stage. The DNA was helically released to form single-stranded DNA at 94°C for 30 s, and primers were bound to the template at 50°C for 30 s and dNTP at 72°C for 80 s, 40 cycles in total. The third stage was the extension stage, which only required 72°C for 7 min and the product was finally preserved at 4°C.

All amplification-enriched products were purified for sequencing using the QIAQUICK gel extract kit (Qiagen, Hillen, Germany), and the amplicons were sequenced on an ABI 3130 Genetic Analyzer (Applied Biosystems, Foster City, CA, United States) as described above.

The results indicated that the VP1 sequences of the seven Tibet strains showed the highest similarity with those of the EV-B75 strain in the GenBank databases, which reached a percentage of as high as 90.95%. We identified all seven strains as EV-B75 based on the enterovirus molecular typing criteria.

### Phylogenetic and Recombination Analysis

For *VP1* phylogenetic analysis, the seven Tibetan EV-B75 sequences generated in this study were combined with all the publicly available VP1 gene sequences in GenBank database with known sampling dates. Maximum likelihood trees were constructed using the GTR + G model as suggested by ModelGenerator0.85 and were implemented in MEGA7 with 1,000 bootstrap replicates. Potential inter-typic and intra-typic recombination was analyzed using SimPlot (version 3.5.1) with a 200-nt window moving with 20-nt steps. To investigate the recombination relationship among the seven Tibetan EV-B75 strains and other enterovirus genotypes, the EV-B75 genome was spliced at the suggested recombination break points. BLAST searches were separately performed using the P2 and P3 coding regions. Closely related enterovirus genome sequences with similarity higher than 85% were selected.

### Test of Neutralization

The Y24 strain was selected as the attack virus in the neutralizing test because it showed the highest titer among the seven Tibetan EV-B75 strains. In total, 112 serum samples were inactivated at 56°C for 30 min, and then diluted at five gradients from 1:4 to 1:1024 (1:4, 1:16, 1:64, 1:256, and 1:1024) for detection. The mixtures comprising a virus culture infection dose (CCID_50_) of 100 (50 μl), human RD cell suspension (100 μl), and serum dilution (50 μl) were then incubated at 36°C in a 5% CO_2_ incubator. With observation for 7 days, the highest dilution of serum that protected 50% of the cultures was recorded based on an EV-like CPE. If the neutralization antibody titer was observed at a dilution higher than 1:8, the serum sample was considered positive and the geometric mean titer was subsequently calculated.

### Assay of Temperature Sensitivity

The temperature sensitivity of the seven plaque-purified EV-B75 strains and two selected control strains (HTYT-ARL-AFP02F/XJ/CHN/2011, showing no temperature sensitivity and HTYPS-QDH11F/XJ/CHN/2011, showing temperature sensitivity) were assayed on monolayer RD cells in 24-well plates. The 24-well plates were inoculated with 50 μL of undiluted virus stocks. Two different incubation plates were used: one was placed at an optimum temperature of 36°C, and the other was placed at the supra-optimal temperature of 39.5°C for virus propagation. After adsorption at 36°C or 39.5°C for 1 h, non-adsorbed virus cultures were discarded and 100 μL of maintenance fluid was added to each well. The 24-well plates were then incubated at 36 and 39.5°C, respectively, and sampled at six time points after infection (4, 8, 16, 24, 48, and 72 h). The CCID_50_ was calculated by the end-point dilution of monolayer RD cells in a 96-well plate at 36°C. Virus isolates that showed a drop of more than 2 log values at different temperatures were considered temperature-sensitive.

## Results

### Global Epidemiology of Novel EV-B75

**FIGURE 1:**
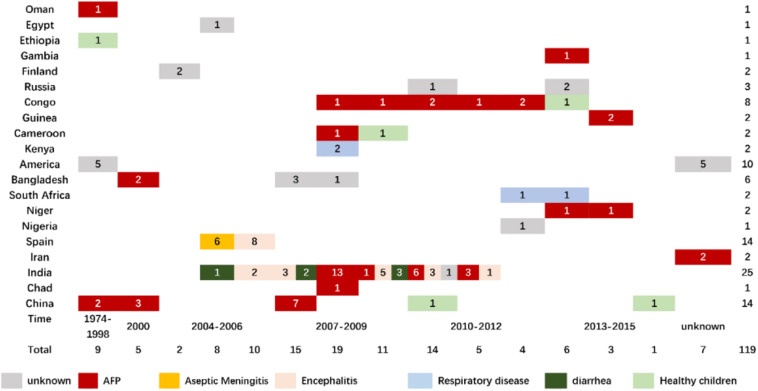
Time, region, and clinical distribution of EV-B75 worldwide.

To date, there are 112 gene sequences in the GenBank database, associated with various clinical manifestations including AFP (Oberste et al., 2004; Bingjun et al., 2008; Rao et al., 2012; Laxmivandana et al., 2013; Sadeuh-Mba et al., 2013; Tao et al., 2014a; Fernandez-Garcia et al., 2017; Maan et al., 2019; Sadeuh-Mba et al., 2019) (47 isolates worldwide), aseptic meningitis (Avellon et al., 2006) (8 isolates in Spain, 2005), encephalitis (Lewthwaite et al., 2010; Kumar et al., 2012) (13 isolates in India, 2006–2010), diarrhea (Rao et al., 2013; Patil et al., 2015) (7 isolates in India, 2005–2011), respiratory diseases (Opanda et al., 2016; Hellferscee et al., 2017) (5 isolates in total), and others (Oberste et al., 2004) (Figure 1). With the addition of the seven Tibetan EV-B75 isolates from this study, no significant or typical temporal and spatial aggregation of EV-B75 was observed worldwide. However, after EV-B75 was molecularly finalized in 2004, the number of isolates was significantly increased in the following years compared with those before finalization. However, the number of related reports was significantly reduced in recent years, possibly due to a lag in reporting. Among these, the case reports of EV-B75 in Spain were relatively concentrated, with one report of six aseptic meningitis-related cases isolated from children’s hospitals in 2005, which was the only report of EV-B75 related to aseptic meningitis worldwide, and another report of the eight encephalitis-associated strains isolated the next year (Cabrerizo et al., 2008). In contrast, there were relatively more isolates in India, with a total of 25. Cases were reported every year from 2005 to 2011, and the types of cases involved were relatively diverse, with AFP (Rao et al., 2012) and encephalitis (Kumar et al., 2012) as the main presentations. Notably, only seven EV-B75 isolates from patients with acute diarrhea worldwide are available in India (Rao et al., 2013; Patil et al., 2015).

Notably, there are only two EV-B75 full-length genome sequences in the GenBank database, one of which is the prototype strain (AY556070, USA-OK85-10362, hereafter referred as strain OK85-10362) and the other is isolated from a child with AFP in Shandong Province, China in 1997 (GQ329840, 102-SD-CHN-97).

### Full-Length Genome Analysis of the Seven Tibetan EV-B75 Strains

Human enteroviruses are small, non-enveloped icosahedral viruses, with a positive-sense single stranded RNA genome, composed of approximately 7,500 nucleotides (nt), including a well-known long open reading frame (ORF) and a newly discovered short second ORF (Guo et al., 2019). The full-length genome sequences of the seven Tibet EV-B75 strains were determined and analyzed. The results showed that the genomes of all strains were 7,425 nucleotides in length. The overall nucleotide composition of the seven strains was as follows: 27.87–28.05% A, 24.08–24.34% T, 23.06–23.35% C, and 24.57–24.69% G. Aligned with the prototype strain of EV-B75 from the United States (OK-10362), all seven strains contained some deletions and insertions, including one deletion at position 16, one deletion at position 24, three deletions at position 107, one insertion at position 572, one deletion at position 7,355, and one insertion at position 7,421 in that order.

The long ORF, franked by a 5′ untranslated region (UTR) and a 3′UTR, was initiated by AUG_744_ and ended at position 7323, totaling 6579 nucleotides in length, encoding a single polypeptide of 2,193 amino acid, which was cleaved into 3 polyprotein precursors P1, P2, and P3, encoding the structural proteins (VP1 to VP4) and non-structural proteins. The second ORF, located in the VI domain of the viral internal ribosomal entry site (IRES) was initiated by AUG_593_ with the WIGHPV (TGG ATT GGC CAT CCG GTG) domain, which is the specific domain of ORF2p and is highly conserved among different species of HEVs; it is important to trigger viral replication and particle release from intestinal epithelial cells (Guo et al., 2019). Non-coding 3′UTRs were 103 nucleotides in length, preceding a long poly(A) tail.

Pairwise comparisons of nucleotide sequences and the deduced amino acid sequences were conducted among the seven Tibetan EV-B75 strains. The full-length genome nucleotide and amino acid similarities were 94.4–99.9 and 99.7–99.9%, respectively, suggesting that the seven Tibetan isolates might have a common origin or ancestor and that their isolation time was relatively concentrated. Furthermore, the nucleotide sequence and amino acid sequence similarities of the VP1 region and P1 region with the EV-B75 prototype strain were 85.3–85.5 and 98.3–98.6%, and 82.3–85.7 and 98.3–100%, respectively. Considering the cutoff value of 75% VP1 similarity for EV typing, these data verified the molecular typing results. As expected, the nucleotide sequence and amino acid sequence similarities of the P2 and P3 regions with the EV-B75 prototype strain were 79.3–81.7 and 97.3–99.0%, and 77.9–82.5 and 95.3–100%, respectively, but they shared a distinctly high identity with other EV-B prototype strains other than EV-B75, suggesting that recombination might occur in these coding regions.

### Phylogenetic Analysis of the Seven Tibet EV-B75 Strains

**FIGURE 2:**
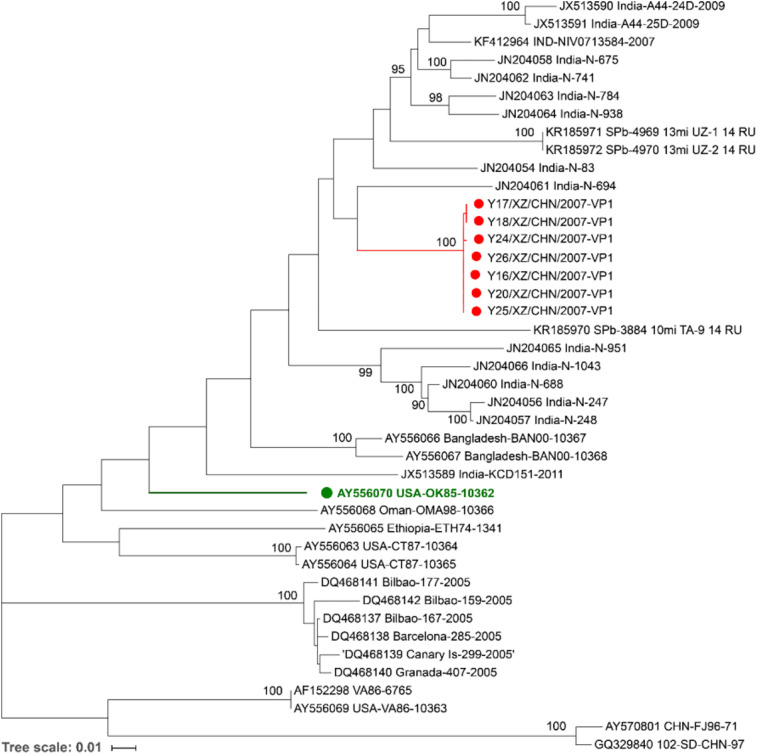
Maximum likelihood phylogenetic tree based on the entire VP1 coding region sequences of EV-B75 available from the GenBank database. The seven Tibetan EV-B75 strains isolated in this study are indicated by red circles, and the prototype of EV-B75 is indicated by a green circle. The scale bars indicate the substitution per site per year. The numbers at the nodes indicate the bootstrap support for the node (percentage of 1,000 bootstrap replicates).

A maximum likelihood phylogenetic tree was constructed for the complete VP1 sequences of the seven Tibet EV-B75 strains and all sequences available in GenBank to study their evolutionary relationships and epidemic patterns (Figure 2).

All the seven Tibet EV-B75 strains were grouped into one branch (with a bootstrap support value of 100%), which verified the high similarity of their VP1 region. They formed a single lineage and were clustered with the Indian strain (JN204061, India-N-694), which was isolated from a patient with AFP in 2007–2009 in India, indicating that they had a closer genetic relationship with the Indian strain. In addition, Shigatse is located at the southwest border of China, adjacent to India. Due to the geographical advantage and the coincidence of onset time and case characteristics, we suspect that the seven Tibetan strains may have originated from India.

Shandong strain (GQ329840, 102-SD-CHN-97) isolated from a patient with AFP in 1997 formed a distinctive lineage with another Chinese strain (AY570801, CHN-FJ96-71) isolated from Fujian Province in 1996, occurred on a separate branch and were grouped separately. Considering that the geographic distance between the two provinces is more than 1000 km, the close genetic relationship possibly reflects a long-distance transmission. This result suggests that more than two different EV-B75 genotypes are existing in China.

**FIGURE 3:**
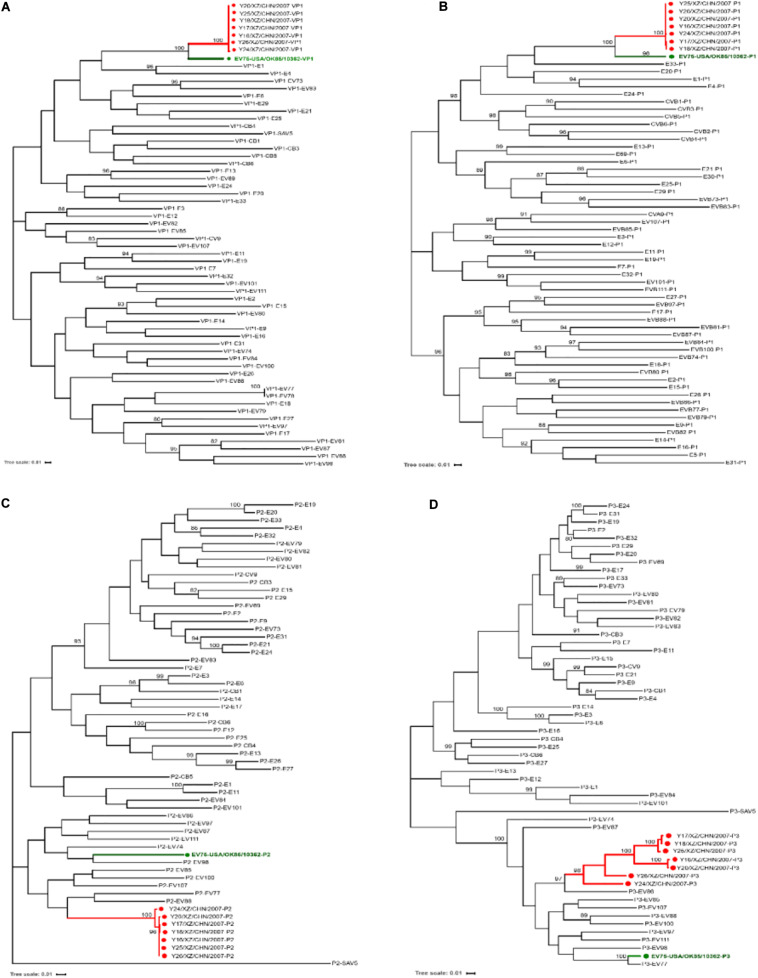
Maximum likelihood phylogenetic tree based on the VP1, P1, P2, and P3 coding regions of the prototype sequence of all EV-B in the GenBank database and the seven Tibet EV-B75 strains in this study. The seven Tibet EV-B75 strains in this study are indicated by red circles, and the prototype of EV-B75 is indicated by a green circle. The scale bars indicate the substitution per site per year. The numbers at the nodes indicate the bootstrap support for the node (percentage of 1,000 bootstrap replicates). Coding sequences of **(A)** VP1, **(B)** P1, **(C)** P2, and **(D)** P3 are shown.

Phylogenetic trees were also constructed based on the entire *VP1*, *P1*, *P2*, and *P3* coding region nucleotide sequences of the seven Tibet EV-B75 strains along with the prototype of EV-B from GenBank database. Consistent with the phylogenetic tree of the P1 coding region (Figure 3B), the VP1 phylogenetic tree (Figure 3A) indicated that the seven Tibet EV-B75 strains formed a single lineage and were clustered with the EV-B75 prototype (OK-10362) as expected, verifying the primary molecular typing results. Unlike the VP1 and P1 phylogenetic trees, the P2 and P3 coding regions phylogenetic trees showed that the seven Tibet EV-B75 strains were clustered together and shared the highest similarity with the prototype strains of EV-B88, EV-B77, and EV-B86, respectively, rather than with the prototype of EV-B75, suggesting the occurrence of recombination between EV-B75 and other EV-B serotypes (Figures 3C,D).

### Recombination Events Between the Seven Tibet EV-B75 Strains and Other EV-B Strains

**FIGURE 4:**
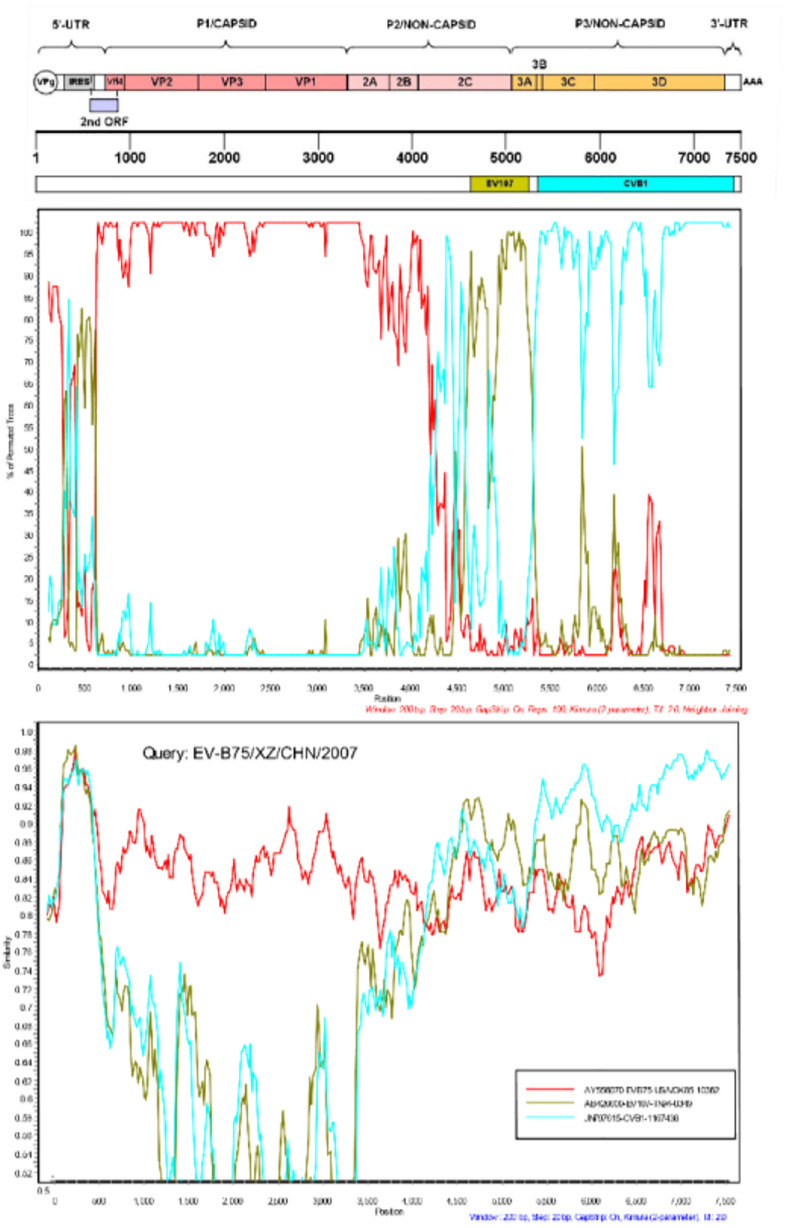
Similarity and boot scanning analysis of the seven Tibetan EV-B75 strains with the prototype of EV-B75, a field EV-107 strain (AB426609-TN-94-0349), and a field CVB1 strain (JN797615-1167438). All seven Tibetan EV-B75 strains were set in a series as a query sequence.

Potential evidence of recombination in the genome of the seven Tibet EV-B75 strains was investigated via similarity plot and boot scanning analysis. Screening of closely related sequences available in GenBank was performed using BLAST from NCBI. The P2 and P3 regions of the seven strains were tested separately. All sequences downloaded from NCBI showing over 85% similarity with the query sequence were used to construct a phylogenetic tree for the P3 region and to set a series in SimPlot (Version3.5.1) if clustered in the same branch and the same serotype. In the P1 region, all seven Tibet EV-B75 strains showed the highest similarity to the EV-B75 prototype strain. In other regions, multiple recombination events were revealed with relatively higher similarities to other EV-B viruses. The similarity plots and boot scanning analysis results indicated that the seven Tibetan EV-B75 strains recombined with the EV-107 strain (AB426609-TN94-0349) at position 4620–5240 and with the CVB1 strain (JN797615-1167438) at position 5340–7425 as shown in Figure 4.

### Seroprevalence of EV-B75 in Tibet

Considering that the first case (Y16) of EV-B75 infection had caused six asymptomatic infections in the same region, we examined the prevalence of EV-B75 among Tibetan population. A total of 112 serum samples from children aged 1–14 years old in Tibet were tested. These were collected from December 2010 to January 2011, including 36 serum samples from Shigatse (27 males and 9 females) and 76 serum samples from Lhasa (44 males and 32 females). Of these, only 3 serum samples were seropositive for EV-B75 (≥1:8) with a total positive rate of 2.61%, of which one was from Lhasa and two were from Shigatse. The geometric mean titer of the positive serum was only 1:4.52. Compared with the seroprevalence rate of EV-71 or even some rarely isolated novel enteroviruses in China, this rate was rather low (Zhu et al., 2010). The low serum positive rate suggested that EV-B75 was limited in the population of Shigatse and Lhasa regions, and that the proportion of people who had been infected or in contact with this serotype was low, due to which there was a limited spread and no epidemic in the region.

### Environmental Tolerance of the Seven Tibetan EV-B75 Strains

**FIGURE 5:**
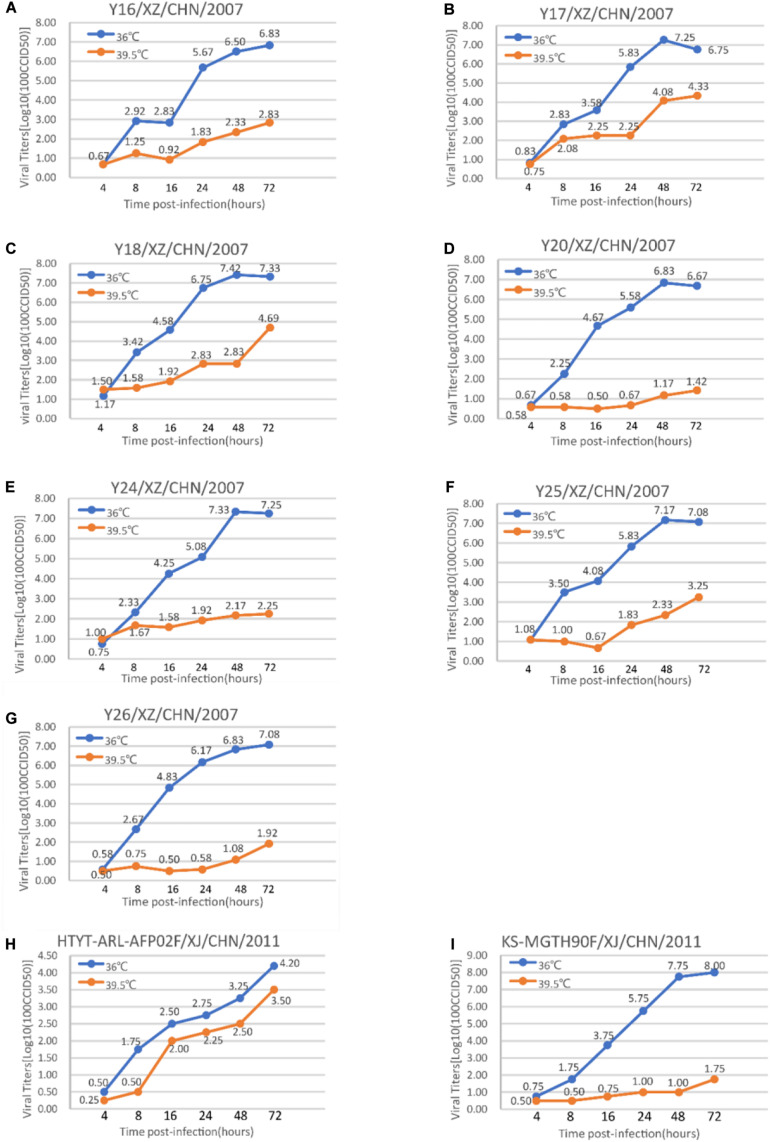
Temperature sensitivity test curves of the seven Tibetan EV-B75 strains. Blue and orange lines represent the growth trends of the viruses on RD cells at 36 and 39.5°C, respectively. The Xinjiang EV-B85 strain (HTYT-ARL-AFP02F/XJ/CHN/2011, showing no temperature sensitivity) and the EV-B106 strain (KS-MGTH90F/XJ/CHN/2011, showing temperature sensitivity) were used as experimental controls. **(A)** Strain Y16/XZ/CHN/2007; **(B)** strain Y17/XZ/CHN/2007; **(C)** strain Y18/XZ/CHN/2007; **(D)** strain Y20/XZ/CHN/2007; **(E)** strain Y24/XZ/CHN/2007; **(F)** strain Y25/XZ/CHN/2007; **(G)** strain Y26/XZ/CHN/2007; **(H)** strain HTYT-ARL-AFP02F/XJ/CHN/2011 (EV-B85); **(I)** strain KS-MGTH90F/XJ/CHN/2011 (EV-B106).

The tolerance of these seven strains to the environment determines their ability to spread. The viral replication ability of these seven EV-B75 strains was compared at different temperatures to determine whether they were sensitive to high temperature (39.5°C). The virus titer of all seven Tibet EV-B75 strains cultured at 36°C for 72 h was higher than 2 logs compared to that at the elevated temperature (39.5°C) (Figure 5). These results indicated that all seven Tibetan EV-B75 strains were temperature-sensitive compared to the EV-B85 strain (Sun et al., 2013) which showed non-temperature sensitivity and the EV-B106 strain (Song et al., 2017) which showed temperature sensitivity.

## Discussion

Based on the sequence similarity among the seven Tibetan EV-B75 strains from Shigatse, Tibet, we speculated that they had the same origin or ancestor. Based on the clustering analysis of phylogenetic trees, combined with the geographical location and clinical features of EV-B75 cases isolated in India in 2007, we speculated that the Tibetan strains were imported from India. Combined with the phylogenetic analysis of Indian strains, the AFP and encephalitis isolates in India are at least clustered into three different clusters, suggesting that EV-B75 has more than one undergoing coevolution, and that its epidemic may be far beyond the currently reported epidemic situation. This further confirmed our speculation that the Tibetan case was imported from India. The Shandong strain and Fujian strain from China were clustered in the phylogenetic tree, separate from other branches, suggesting that there might be two existing branches of EV-B75 in China.

We also investigated the seroepidemiology of EV-B75 in Tibet, China and found a very low seropositive rate, which might indicate the seven strains isolated in Tibet, China in 2007 to be the first batch of EV-B75 serotype isolates found, and that these have not widely circulated in the population. This strengthens the hypothesis that the seven Tibet EV-B75 strains were from India. The results of temperature sensitivity also suggested that the transmissibility of EV-B75 was limited and that it was not resistant to high temperature, which also contributed to the relatively limited spread of the Tibetan strain.

Although the transmission range of EV-B75 in Tibet was speculated to be limited in this study, EV-B75 has two existing circulations in China, and the transmission potential of the novel enterovirus in China cannot be underestimated. Further, due to the seroprevalence of other novel enteroviruses reported in China, even though there are only a few isolates, the seropositive rate of novel enteroviruses in China is not low, indicating the potential for an outbreak (Hu et al., 2014; Zhang et al., 2014; Fan et al., 2015; Song et al., 2017; Han et al., 2018; Huang et al., 2018; Lu et al., 2020; Xiao et al., 2020). The proportion of people who have been exposed to the virus is relatively high, and it thus has great transmission potential in China.

Recombinant analysis revealed that EV-B75 strains were extensively recombined with other EV-B enteroviruses in the P2 and P3 regions, and that recombination was indeed common in enteroviruses, especially in these two regions. This serotype might thus undergo gene sequence exchange and evolution with other enteroviruses in a human or environmental epidemic; further, this genotype has existed in the human population for some time and has been epidemic for some time, rather than being a new-born serotype. Thus, it needs to be monitored.

At present, the clinical symptoms of EV-B75 serotype-related strains in GenBank are relatively diverse, involving AFP, aseptic meningitis, encephalitis, acute diarrhea, and some flu-like symptoms, and these have been collected from feces, cerebrospinal fluid, and pharyngeal swabs. Although there are currently 112 EV-B75-related cases, limited studies have targeted EV-B75 as the causative agent. The relationship between pathogens and diseases is far from being conclusive and needs to be proved using more detailed epidemiological information, more full-length genome sequences, and observations of pathological changes in animals. In this study, we explored the possible sources of this outbreak of EV-B75 in Tibet, investigated the population prevalence of EV-B75 and the transmission ability of these strains in Tibet, and answered some epidemiological questions about this outbreak. However, due to the limited case information in GenBank, the gene sequences with different clinical manifestations were not compared. Afterward, based on the existing experiments, we will conduct animal experiments to study the dynamic distribution characteristics and pathological changes of the virus in different tissues and organs of animals after the attack, in order to try to explain the potential relationship between the pathogen and the disease. Our study on EV-B75 is just a stepping stone, and additional studies are needed to supplement.

China is a country with a long border shared with many countries, which are facing great pressure on the health system. In addition, with the progress of globalization and increasingly frequent communication between China and other countries, the pressure on the health system at China’s border areas will also increase. For instance, in 2011, China experienced an outbreak caused by wild poliovirus type I imported from Pakistan, affecting 10 young children and 11 adults, resulting in 2 deaths (Luo et al., 2013). As a lesson learned from these cases, more attention should be paid for the prevention of epidemics along the border.

## Data Availability Statement

The full-length genomic sequences detected in this study have been deposited to China Virus Identification Net under accession numbers CVIN_AA002552–CVIN_AA002558 and to GenBank under accession numbers MW183133–MW183139.

## Author Contributions

KZ, YZ, GW, and WX: conceptualization. KZ, YZ, ZH, JX, HL, YS, DY, DW, and SZ: formal analysis. MH: resources. KZ: writing—original draft preparation. YZ: writing—review and editing and funding acquisition. YZ and GW: project administration. All authors have read and agreed to the published version of the manuscript.

## Conflict of Interest

The authors declare that the research was conducted in the absence of any commercial or financial relationships that could be construed as a potential conflict of interest.
